# PFC/PFAS concentrations in human milk and infant exposure through lactation: a comprehensive review of the scientific literature

**DOI:** 10.1007/s00204-025-03980-x

**Published:** 2025-02-22

**Authors:** Neus González, Jose L. Domingo

**Affiliations:** Laboratory of Toxicology and Environmental Health, School of Medicine, Universitat Rovira I Virgili, San Llorens 21, 43201 Reus, Catalonia Spain

**Keywords:** Perfluorinated compounds (PFC), Per- and polyfluoroalkyl substances (PFAS), Human exposure, Breast milk, Daily intake

## Abstract

Per- and polyfluoroalkyl substances (PFAS), previously known as perfluorinated compounds (PFC), are a group of synthetic chemicals widely used over the past decades. Their extensive application, combined with their environmental persistence, has contributed to their ubiquitous presence in the environment and the associated toxicological risks. Regarding humans, blood serum testing remains the primary method for biomonitoring PFAS exposure, while breast milk has also been used due to the transfer of these substances from mothers to infants during lactation. This paper aims to review the scientific literature (using PubMed and Scopus databases) on PFAS concentrations in the breast milk of non-occupationally exposed women. Where available, the estimated daily intake of these compounds by breastfeeding infants is also examined. The reviewed studies are categorized by continent and country/region, revealing a significant lack of data for many countries, including both developed and developing nations. The findings indicate substantial variability in PFAS concentrations, influenced by factors such as geographic location, sampling year, and the specific PFAS analyzed. Among the identified compounds, perfluorooctane sulfonate (PFOS) and perfluorooctanoic acid (PFOA) are most commonly detected, along with perfluorohexanesulfonic acid (PFHxS) and perfluorononanoic acid (PFNA), being the only PFAS with regulated maximum levels in certain foodstuffs. Most studies were conducted before the implementation of the current (updated) tolerable weekly intake (TWI) values for these substances. Consequently, the majority reported a low health risk for breastfeeding infants, even in high-intake scenarios. Nevertheless, biomonitoring studies are urgently needed in countries with limited or no data, and new investigations should assess whether current estimated intakes exceed the updated TWI. Special focus should be given to rural and industrial areas where exposure levels remain poorly understood.

## Introduction

Perfluorinated compounds (PFC) are a group of synthetic chemicals characterized by chains of carbon atoms, which are fully or partially fluorinated. Around the early 2000s, PFC were renamed as per- and polyfluoroalkyl substances (PFAS), but the term PFAS was more widely adopted over the following decade (Langenbach and Wilson 2021; Evich et al. 2022). PFAS, which include a wide group of compounds, contain carbon chains with fluorine atoms attached, being the two primary types, perfluoroalkyl substances and polyfluoroalkyl substances, hydrophobic and lipophobic (Kwiatkowski et al. 2020). PFAS are synthesized by two processes: direct fluorination and oligomerization, being also thermally and chemically stable (Kim et al. 2015; Leung et al. 2023). The term PFAS was introduced to better categorize the diverse and very extensive group of synthetic chemicals containing fluorinated carbon chains, among which substances such as perfluorooctanoic acid (PFOA) and perfluorooctane sulfonate (PFOS) were already well known given their environmental persistence and potential health risks. Nowadays, PFAS is the term used by international and national environmental/regulatory agencies/organizations, as well as by most scientists.

Due to their durability, resistance to heat, water, and oil, as well as non-stick properties, PFAS have been widely used in industrial and consumer products (Glüge et al. 2020; Meegoda et al. 2020). This widespread and continued use for years, together with their persistent nature, is responsible for their environmental occurrence (Cousins et al. 2020; Zhang et al. 2022; Lohmann and Letcher 2023) and their potential human health risks (Brase et al. 2021; Panieri et al. 2022). PFAS may enter the environment through industrial discharges, landfill leachate, wastewater treatment plant effluents, and the breakdown of consumer products, being found in air, water, soil, and sediments (Domingo and Nadal 2019; Hu et al. 2021a, b; Mei et al. 2021; Post 2021; Zhang et al. 2023; Ohoro et al. 2024). Some well-known PFAS, such as PFOS and PFOA, can bioaccumulate in the food chain, particularly in aquatic organisms, being a potential source of human exposure, mainly through the consumption of fish and seafood (Domingo 2012; Domingo and Nadal 2017). The use of food-contact packaging applications, non-stick cookware, firefighting foams, and cosmetics, among others, are also potential sources of human exposure to PFAS (Pelch et al. 2019; Curtzwiler et al. 2021; Holder et al. 2024; Wolf et al. 2024).

Regarding the toxicity of PFAS, laboratory animal studies have demonstrated that certain PFAS, mainly the most investigated PFOS and PFOA, cause toxic effects in rodents, impacting multiple organ systems. Key findings include liver toxicity, endocrine disruption, developmental and reproductive toxicity, and potential carcinogenicity. Lau et al. (2007) reported that PFOA and PFOS disrupt lipid metabolism, resulting in hyperlipidemia and fatty liver disease. In turn, Chang et al. (2008) showed that PFOS interferes with thyroid hormone regulation, leading to hypothyroxinemia in rats and mice. Moreover, prenatal exposure to some PFAS has been linked to reduced fetal growth, delayed development, and skeletal malformations in rodent models, while exposure to PFNA impairs fertility, reduces litter size, and disrupts ovarian function (Das et al. 2015). In addition, PFOS induced liver, pancreatic, and testicular tumors in rodents (Butenhoff et al. 2012). These effects are thought to occur through mechanisms including activation of peroxisome proliferator-activated receptors (PPARs) and disruption of hormonal pathways. On the other hand, in vitro studies on PFAS toxicity have revealed their ability to disrupt cellular processes across various systems (Behr et al. 2020). PFOA and PFOS induce cytotoxicity in human and animal cell lines, leading to reduced viability and increased apoptosis, while PFOS exposure has been reported to cause oxidative stress, increasing reactive oxygen species (ROS) levels, causing oxidative damage, and impairing mitochondrial function (Elumalai et al. 2023). Additionally, it has been shown that PFAS modulate immune cell function, reducing cytokine production and impairing immune responses (Liang et al. 2022), whereas some PFAS may also perturb neurodevelopmental processes in vitro (Carstens et al. 2023).

**Fig. 1:**
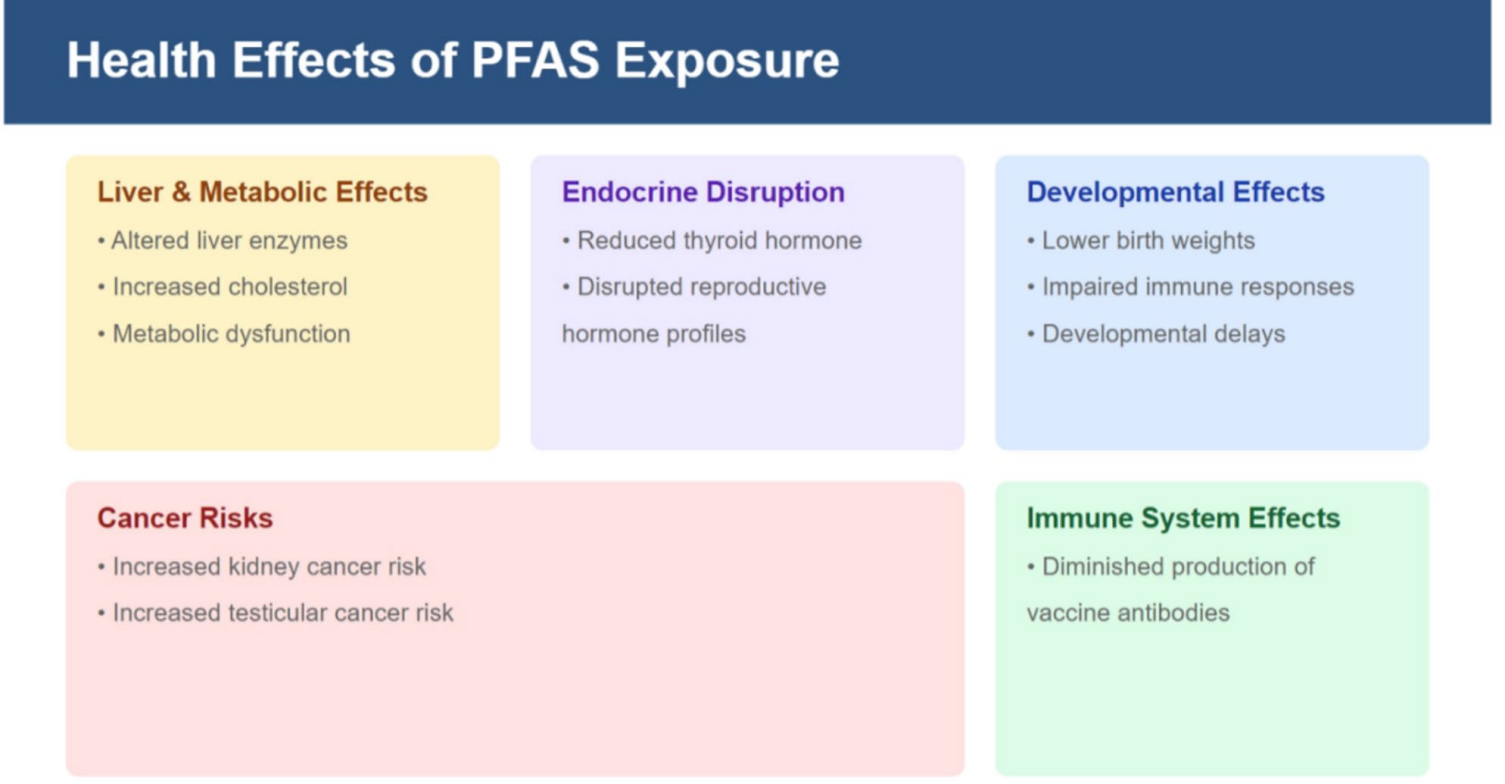
Summary of key adverse health effects associated with exposure to PFAS

It has been reported that PFAS can cause endocrine disruption since they may interfere with hormone function, potentially affecting thyroid function and reproductive health (Ding et al. 2020; Rickard et al. 2022), as well as immunotoxicity, considering that some PFAS can reduce immune response, affecting the ability of the body to fight against infections (Liang et al. 2022; Sonne et al. 2023; Post et al. 2024). Evidence of a diminished production of vaccine antibodies caused by some PFAS, mainly PFOA, PFOS, and PFHxS, has been provided by epidemiological investigations (Abraham et al. 2020; Crawford et al. 2023). Moreover, exposure to PFAS has been related to increased cholesterol levels, liver damage, and potential increased risks of metabolic diseases (Sunderland et al. 2019; Wen et al. 2023; Wu et al. 2023), while exposure to some compounds like PFOA has been even associated with an increased risk of certain cancers (Steenland and Winquist 2021; Boyd et al. 2022). Key adverse health effects associated with exposure to PFAS are summarized in Fig. 1.

On the other hand, PFAS can also harm wildlife, especially aquatic organisms and mammals (Chen et al. 2021; Cheng et al. 2022; Pandelides et al. 2023), which in turn may accumulate these substances through the food chain (Pan et al. 2021; Sun et al. 2022; Hopkins et al. 2023). The Stockholm Convention on Persistent Organic Pollutants (POPs Convention) has recognized the significant risks posed by certain PFAS compounds, specifically PFOS, PFOA, and PFHxA, by adding them to its list of regulated substances requiring prohibition, restriction, or minimization (UNEP 2024). Given the mounting evidence of potential health risks to the general population from chronic environmental and dietary PFAS exposure, human biomonitoring of these persistent chemicals has emerged as a critical public health surveillance priority.

The persistent presence of PFAS in the environment, food and drinking water, poses significant health risks to populations worldwide, making systematic human biomonitoring of these chemicals a public health priority. In recent years, several studies have quantified the concentrations of various PFAS in humans through both invasive and non-invasive biomonitoring methods, primarily in blood, breast milk, urine, and occasionally in hair and nails. Like many other POPs (Schuhmacher et al. 1999; Wang et al. 2013; Aylward et al. 2014; Thomas et al. 2017), blood serum testing remains the most widely used biomonitoring method. This is due to the tendency of PFAS to bind to proteins in the blood, where they can persist for extended periods, providing a reliable indicator of exposure (Ericson et al. 2007; Haug et al. 2011; Wu et al. 2015; Ho et al. 2022; McAdam and Bell 2023). The aim of the present paper was to update the available scientific literature on the concentrations of PFAS in breast milk, a non-invasive biomonitoring medium, among non-occupationally exposed women. As PFAS can be transferred from mothers to infants through breast milk, when data were available, the daily intake of these substances by breastfeeding infants has also been reviewed.

### Search strategy

The scientific databases Scopus (https://www.scopus.com) (accessed on August 22, 2024) and PubMed (https://pubmed.ncbi.nlm.nih.gov/) (accessed on August 22, 2024) were used for the search of articles directly related with the main topic of the present review. The search included all papers cited in these databases without restrictions on the date of publication of the articles. For the search, the following terms/keywords—and their combinations—were used: perfluorinated compounds, PFC, perfluoroalkyl substances, polyfluoroalkyl substances, PFAS, human milk, breast milk, breastfeeding infants, and infants’ exposure.

## Data belonging to studies on the topic conducted around the world, classified by continents and countries/regions

### ASIA

#### China

So et al. (2006) collected breast milk samples from 19 volunteers in Zhoushan, in which the concentrations of various PFC were measured. The health risks for infants—via consumption of mothers' breast milk—were also assessed. Among the analyzed PFC, perfluorobutanesulfonic acid (PFBS) and perfluorohexanoic acid (PFHxA) were not detected in any sample, while perfluorohexanesulfonic acid (PFHxS), PFOS, PFOA, perfluorononanoic acid (PFNA), perfluorodecanoic acid (PFDA), and perfluoroundecanoic acid (PFUnDA) could be quantified in all samples, with PFOS (range 45–360 ng/L) and PFOA (range 47–210 ng/L) being the dominant PFC. The highest levels of other analyzed PFC were the following: 100 ng/L for PFHxS, 62 ng/L for PFNA, 15 ng/L for PFDA, and 56 ng/L for PFUnDA. With respect to the intakes of PFC for children via breast milk, those for PFOS and PFOA were 0.030 and 0.017 μg/kg bw/day, respectively. Only in one of the 19 analyzed samples, the hazard index (HI) for PFOS was greater than the unity (HI > 1). Liu et al. (2010) determined the levels of 10 PFC in 24 pooled milk samples collected from 1237 individuals living in 12 provinces of China. The daily intakes of PFC by breastfeeding infants were also assessed. Only six PFC (PFHxS, PFOS, PFOA, PFNA, PFDA, and PFUnDA) were detected, with the highest frequencies of detection corresponding to PFOS and PFNA (both 100%), followed by PFOA and PFDA (both 87.5%), and PFHxS and PFUnDA (both 83%). PFOS (median level 49 ng/L; range 6–137 ng/L) and PFOA (median level 34.5 ng/L; range < LOD–814 ng/L) were the predominant PFC in all samples. The total levels of the six detected PFC ranged between 26 and 1252 ng/L, with a median of 133 ng/L. Regarding the estimated daily intake (EDIs) for breastfeeding infants, the median and the highest values of total PFC were 17.2 and 129.1 ng/kg bw/day. In the Shanghai region (one of the examined regions in that survey) mothers and children had a high exposure to PFC. In a subsequent study performed by the same research group (Liu et al. 2011), the concentrations and profiles of PFC in maternal blood, cord blood, and human breast milk samples, collected in Jinhu county, Jiang Su province, were determined to characterize the exposure of newborns to PFC during the periods of gestation and lactation. Regarding breast milk, PFOS, PFOA, PFNA, PFDA, and PFUnDA showed the highest detection frequencies, which ranged between 72 and 100%. In general, PFC levels in milk were lower than the matched serum concentrations. The highest median levels in maternal serum corresponded to PFOA (1.264 × 10^3^ ng/L) and PFOS (2.922 × 10^3^ ng/L), while in milk samples the median concentrations of PFOA and PFOS were 121 and 42 ng/L, respectively. Comprehensive partition ratios of PFC through placental barrier and lactations were found, indicating a high transport rate for PFOA. Postnatal exposure of PFC through lactation was found to be higher than prenatal exposure, which was especially noted for PFOA.

On the other hand, in Hangzhou, Jin et al. (2020) carried out a study aimed at characterizing the presence of PFAS and their concentrations in breast milk from 174 women, as well as to estimate the PFAS exposure for infants at birth. A total of 16 PFAS were detected, with the levels of total PFAS (∑PFAS) ranging between 9.0 and 1860 ng/L, with a mean of 205 ng/L. The highest frequencies of detection corresponded to PFOA (100%), 6:2 chlorinated polyfluorinated ether sulfonate (6:2 Cl-PFESA) (100%), and PFUnDA (84%), with more than one-half of the samples containing perfluorobutanoic acid (PFBA), perfluoropentanoic acid (PFPeA), long-chain perfluorocarboxylic acids (C_9_-C_11_ PFCAs), PFOS, 1H,1H,2H,2H-Perfluoro-1-decanol (8:2 FTOH), and 1H,1H,2H,2H-Perfluoro-1-Dodecanol (10:2 FTOH). The highest mean concentration corresponded to PFOA (87 ng/L), followed by PFHxA (41 ng/L), and 6:2 Cl-PFESA (28 ng/L), while that of PFOS was 25 ng/L. Increased levels of PFOA, PFNA, PFDA, and 6:2 Cl-PFESA in milk were associated with decreased infant's length gain rate. The EDIs of PFOA and PFOS for infants were lower than the respective tolerable daily intakes (TDIs). Zheng et al. (2022) analyzed the concentrations of 21 PFAS in maternal serum, cord serum, and breast milk of 60 sets of matched maternal–neonatal samples collected in Mianyang, Sichuan Province. The pathways of transplacental transfer during gestation, as well as breastfeeding transfer during lactation, were compared. The prenatal and postnatal exposure of newborns to PFAS were also characterized. Twelve PFAS could be detected in the three analyzed matrices. With respect to breast milk samples, PFOA, PFOS, and 6:2 fluorotelomer phosphate diester (6:2 diPAP) were the predominant PFAS, with mean values of 87, 53, and 35 ng/L, respectively. The highest EDIs corresponded to PFOA, 34.5 ng/kg bw/day, PFOS, 9.7 ng/kg bw/day, and 6:2 diPAP, 11.0 ng/kg bw/day. The results of that study showed that, in general, postnatal exposure to PFAS via breastfeeding was higher than the prenatal exposure “in utero*,*” with legacy compounds such as PFOA and PFOS causing a greater exposure to newborns than emerging PFAS. On the other hand, to assess the exposure of breastfed infants to toxic organic pollutants emitted by a municipal solid waste incinerator located in Zhejiang Province, Xu et al. (2022) performed a cross-sectional study aimed at evaluating the concentrations of polychlorinated biphenyl (PCB), dioxins and furans (PCDD/Fs), and PFAS in mothers’ breast milk as well as to evaluate infants’ exposure. Among the 21 analyzed PFAS, only ten were detected, being 100% the detection frequencies of PFBA, PFOA, and PFUnDA, while those of the rest of detected compounds ranged between 3 and 93%. The median total PFAS level was 250 ng/L (range 151–833 ng/L). In relation to the distribution of the PFAS, the highest percentages corresponded to PFOA, PFOS, and perfluorotetradecanoic acid (PFTeDA), with 46%, 15%, and 12%, respectively. In general, there were high burdens of total PFAS, in relation to women of other countries. However, their concentrations were comparable to those reported in other industrial regions of China. The infant’s mean EDIs of PFOS, PFOA, and PFNA were 5.8, 17.9, and 1.7 ng/kg bw/day, respectively. In a more recent study, which was also focused on determining the concentrations of PFAS in breast milk, Chen et al. (2024) analyzed samples of 324 women of Yingcheng, Hubei Province. With the obtained results, the exposure of infants through breast milk was also assessed. Among 23 analyzed PFAS, the highest median concentrations corresponded to PFOS (200.7 ng/L), PFOA (63.5 ng/L), and PFHxS (25.2 ng/L). For infants, the median EDIs were 25.1 (PFOS), 7.9 (PFOA), and 3.2 (PFHxS) ng/kg bw per day. A summary of the studies conducted in China, in which the concentrations of PFAS were determined in human milk samples, is presented in Table 1.

**Table 1:** Studies conducted in China, in which the concentrations of PFAS in human milk samples were determined

Area/region/city	PFC/PFAS included in the study	Occurrence and concentrations of PFOS and PFOA	Occurrence (detection frequency, DT) and concentrations of other PFC/PFAS	Daily intakes of PFC/PFAS through milk by breastfeeding infants	References
Zhoushan	PFBS, PFHxS, PFOS PFHxA, PFHpA, PFOA, PFNA, PFDA, PFUnDA, 8:2 FTCA, 8:2 FTUCA	PFOS (range 45–360 ng/L) and PFOA (range 47–210 ng/L)	The highest levels were the following: 100 ng/L for PFHxS, 62 ng/L for PFNA, 15 ng/L for PFDA, and 56 ng/L for PFUnDA	The EDIs by children of PFOS and PFOA, via breast milk, were 0.030 and 0.017 μg/kg bw/day, respectively	So et al. (2006)
12 Chinese Provinces	PFHxS, PFOS, PFOA, PFNA, PFDA, PFUdA, PFHpS, PFPeA, PFHpA, PFHxA	100% of detection for PFOS and PFOA. PFOS (median level 49 ng/L; range 6–137 ng/L) and PFOA (median level 34.5 ng/L; range < LOD-814 ng/L)	The total levels of the 6 detected PFC (PFHxS, PFOS, PFOA, PFNA, PFDA and PFUdA) ranged between 26 and 1252 ng/L, with a median of 133 ng/L	The median and the highest values of total PFC were 17.2 and 129.1 ng/kg bw/day	Liu et al. (2010)
Jinhu county, Jiang Su Province	PFOA, PFHxS, PFDS, PFPeA, PFHxA, PFDoA, PFTrDA, PFOS, PFNA, PFDA, PFUdA	Median concentrations of PFOA and PFOS: 121 and 42 ng/L, respectively	PFNA, PFDA and PFUdA were detected at 100%, 78% and 72% of the samples, respectively	Not reported	Liu et al. (2011)
Hangzhou	16 PFAS	Occurrences: PFOS, 50% and PFOA, 100%. Mean concentrations: PFOA, 87 ng/L, and PFOS, 25 ng/L	DTs: D6:2 Cl-PFESA (100%), PFUnA (84%), and more than 50% for PFBA, PFPeA, C_9_-C_11_ PFCAs,, 8:2 FTOH, and 10:2 FTOH. PFHxA (41 ng/L) and 6:2 Cl-PFESA (28 ng/L)	The EDIs of PFOA and PFOS for infants were lower than the respective tolerable daily intakes (TDIs)	Jin et al. (2020)
Mianyang, Sichuan Province	21 PFAS	Mean concentrations: PFOS, 53 ng/L and PFOA 87 ng/L	6:2 diPAP was one of the most detected PFAS, with a mean level of 35 ng/L	The highest EDIs corresponded to PFOA, 34.5 ng/kg bw/day, PFOS, 9.7 ng/kg bw/day, and 6:2 diPAP, 11.0 ng/kg bw/day	Zheng et al. (2022)
Zhejiang Province (near a municipal solid waste incinerator)	21 PFAS	The detection frequency of PFOA was 100%. The specific concentrations of PFOA and PFOS were not reported	Only 10 PFAS were detected, with PFBA and PFUdA having detections of 100%. The median total PFAS level was 250 ng/L (range 151–833 ng/L)	The infant’s mean EDIs of PFOS, PFOA, and PFNA were 5.8, 17.9, and 1.7 ng/kg bw/day, respectively	Xu et al. (2022)
Yingcheng, Hubei Province	23 PFAS	Median concentrations: PFOS (200.7 ng/L), PFOA (63.5 ng/L),	Median concentration of PFHxS: 25.2 ng/L	The median EDIs for infants were 25.1 (PFOS), 7.9 (PFOA) and 3.2 (PFHxS) ng/kg bw/day	Chen et al. (2024)

#### South Korea

Kim et al. (2011) conducted a study aimed at examining the distribution of various PFC among maternal serum, cord serum, and breast milk of residents in Seoul. In samples of breast milk, only PFOA, PFHxS, and PFOS were detected at frequencies of 47%, 88%, and 100%, respectively, while perfluoroheptanoic acid (PFHpA), perfluorododecanoic acid (PFDoDA), PFBS, perfluorododecane sulfonate (PFDS), and perfluorooctanesulfonamide (PFOSA) could not be detected in any sample. PFOS and PFOA were the main contributors (94%) to the total PFC concentrations in maternal breast milk, with mean levels of 61 and 41 ng/L, respectively, notably higher than the mean level of PFHxS (7.2 ng/L). Based on these results, the mean EDIs for Korean infants during the first 6 months of life were found to be 4.7 ng/kg bw/day for PFOA, 0.8 ng/kg bw/day for PFHxS, and 6.9 ng/kg bw/day for PFOS. Another study on the same topic was carried out by Kang et al. (2016), who measured the concentrations of various PFAS in breast milk of Korean women living in four regions of the country. The potential associated risks of PFAS for breastfed infants were also assessed. Among the 17 analyzed compounds, the highest percentages of detection corresponded to PFOA and PFOS (both 98.5%), with medians of 72 and 50 ng/L, respectively, while PFPeA, PFHxA, and PFHpA were detected at percentages of 81.8, 70.8, and 67.4%, being their median values of 58, 47, and 28 ng/L, respectively. The frequencies of detection for PFNA, PFBS, PFDS, PFUnDA, PFDoDA, and PFHxS were all lower than 50% (range 11.7–42%). For breastfed infants, the EDIs for PFOA and PFOS were in the range 3.4–11 ng/kg bw/day, suggesting that exposure to these compounds poses minimal health risks. In turn, Lee et al. (2018) determined the levels of 16 PFAS in samples of breast milk collected from 127 Korean mothers, who were recruited from the cities of Seoul, Pyeongchang, Ansan, and Jeju. The relationships between the levels of PFAS and various demographic parameters were also examined. Moreover, the daily intakes of PFAS, via consumption of breast milk, were estimated. PFOS was found in 100% of samples, while other PFAS were also detected at comparatively high/moderate frequencies: PFOA (88%), PFUnDA (86%), PFNA (63%), PFHxA (40%), PFHS (35%), and PFHpA (26%). In contrast, the remaining analyzed compounds had detection rates lower than 5%. The highest levels in breast milk samples corresponded to PFOS (mean: 57.3 ng/L; range 14.8–380 ng/L) and PFOA (mean 55.6 ng/L; range < 10–657 ng/L, being 188 ng/L (range 31.7–1004 ng/L) the mean concentration of ΣPFAS. It was observed that the levels of PFAS in breast milk were significantly correlated with maternal age, body mass index (BMI), and parity. When exposure to PFAS through consumption of breast milk by infants was evaluated, the mean EDI of ΣPFAS was 17.5 ng/kg bw/day, 90 days after birth, while those of PFOS and PFOA (also 90 days after birth) were 5.36 and 4.75 ng/kg bw/day, respectively. Recently, the same research group (Kim et al. 2023) reported the results of a survey whose main objectives were to determine (and/or to compare) the concentrations of 14 PFAS in breast milk, to compare the time-course trend, as well as to establish which were the potential influencing factors. Samples were collected proportionally to the regional fertility rate in all regions of Korea. PFOS, PFOA, and PFDA were found in all the analyzed samples, being their median levels: 50, 100, and 7 ng/L, respectively. PFHxS (detection rate: 87.4%), PFNA (87.0%), and PFHxA (72.9%) had median levels of 55, 9, and 48 ng/L, respectively. In contrast, PFBS and PFDS could not be detected in any sample, while PFPeA, PFHpA, PFDoDA, and PFTeDA were only found in a few samples (detection range 0.5–7.25%). In relation to the trend in the concentrations of PFAS in breast milk, no significant variation in mean PFOS level was observed in a period of 12 years, but the mean PFOA level increased approximately three times. The main factors related to PFAS concentrations were to live in non-metropolitan areas, the BMI, neonatal age, as well as the consumption and frequency of some food, mainly fish and seafood. For neonates, the EDIs of PFHxS, PFOS, PFHxA, PFOA, PFNA, PFDA, ∑perfluorosulfonic acids (PFSAs), ∑perfluoroalkyl carboxylic acids (PFCAs), and total PFAS were 0.89, 0.81, 0.65, 1.57, 0.13, 0.11 and 3.87 ng/kg bw/day, respectively.

#### Jordan

Al-Sheyab et al. (2015) determined the concentrations of PFOS and PFOA in 79 samples of human milk from breastfeeding women (also local fresh cow milk) collected in northern Jordan. PFOA could be detected in all samples (mean: 143.64 ng/L, range 24–1220 ng/L), while PFOS was found in 74 samples (mean: 34.78 ng/L, range < 10–178 ng/L). The mean levels of both compounds were significantly higher in samples of older women, while the mean concentrations of PFOA were much higher in multiparas.

#### Other Asian countries

On the other hand, Tao et al. (2008a) conducted a wide study on the topic, in which the concentrations on nine PFC (PFOS, PFOA, PFHxS, PFNA, PFBS, PFHpA, PFDA, PFUnDA, and PFDoDA) were measured in human milk from seven Asian countries (Japan, India, Malaysia, Philippines, Indonesia, Vietnam, and Cambodia). PFOS was the most detected compound in the analyzed samples, being found in 100% of the samples from all countries, excepting India (85%). PFHxS and PFOA were also detected in a high number of samples. The average EDI of total PFC was 18.2 ± 14.3 ng/kg bw/day, for the seven countries included in the study. Based on the highest concentrations detected, the average EDIs were 64.6, 41.3, and 88.7 ng/kg bw/day, for PFOS, PFOA, and total PFC respectively.

### Europe

#### Nordic countries

In Norway, Thomsen et al. (2010) investigated the elimination rates of various groups of POPs during lactation. Seven PFC (PFOA, PFNA, PFDA, PFUnDA, PFHxS, perfluoroheptanesulfonic acid (PFHpS) and PFOS) were included in that survey. For it, the levels of the selected compounds were measured in longitudinally collected breast milk samples from Norwegian mothers. With respect to PFC, only PFOS and PFOA could be quantified, being their median (range) concentrations: 110 (28–360) ng/L and 50 (16–190) ng/L, respectively. The EDIs were 61 and 112 ng/day for the infants at the beginning of the breastfeeding, with significant depuration rates of 3.8 and 7.8% per month, for PFOS and PFOA, respectively. In a subsequent study conducted by the same research group, the intakes of PFC from food, drinking water, dust ingestion, and inhalation were determined for a group of Norwegian women. PFC exposure of infants through consumption of breast milk was also estimated for approximately one-half of the volunteers (Haug et al. 2011). Among the 11 analyzed PFC, PFOS (mean: 93 ng/L, range 40–250 ng/L) and PFOA (mean: 76 ng/L, range < 18–830 ng/L) showed, by far, the highest concentrations in samples of breast milk. The median estimated intake of both compounds for six months old infants ranged between 8.7 and 9.1 ng/kg bw/day, and between 4.3 and 4.9 ng/kg bw/day, for PFOS and PFOA, respectively. For these infants, breast milk was found to be the main source of PFC exposure, with the maximum intakes being relatively close to the estimated TDIs for lifelong exposure. In another investigation performed by the same research group, the levels of PFOS and PFOA were measured in samples of breast milk to assess the possible association of these PFAS with different child neuropsychological development, which was assessed at 6, 12, and 24 months (Forns et al. 2015). The median concentrations of PFOS and PFOA in breast milk were 110 and 40 ng/L, respectively. The results of the neuropsychological evaluations did not show any association between perinatal PFOS and PFOA exposure and cognitive, psychomotor, and behavioral development. On the other hand, Iszatt et al. (2019) assessed whether various groups of environmental toxicants (polybrominated diphenyl ethers (PBDEs), PCBs, PFAS, and organochlorine pesticides (OCPs)) in breast milk of Norwegian mothers could affect the composition and function of the infant gut microbiome at one month. PFOS and PFOA were the measured PFAS in breast milk, showing, at one-month post-partum, the following mean concentrations: 126.70 (range 22.99–370.63) ng/L and 57.60 (range 2.19–182.58) ng/L, respectively. At that time, PFOS, but not PFOA, was associated with less microbiome diversity. In turn, again in the line of investigating the possible relationship of the concentrations of certain POPs in breast milk with potential adverse effects on normal child development, the same research group (Lenters et al. 2019) assessed the association of early-life exposure to PBDEs, PCBs, OCPs, and PFOS and PFOA, with the risk of attention-deficit/hyperactivity disorder (ADHD) in a birth cohort of 2606 Norwegian mother–child pairs. The concentrations of the POPs were measured in breast milk samples, and postnatal exposures were estimated using a pharmacokinetic model, in the first two years of life. Regarding specifically the two analyzed PFAS, a positive association between the median level of PFOS (117 ng/L) in breast milk and the risks of ADHD was noted. However, it was sex specific, being stronger and significant only in girls.

In Sweden, Kärrman et al. (2007) determined the concentrations of PFC in breast milk of women from Uppsala, and their exposure through lactation. Among the analyzed PFC, only five (PFOS, PFHxS, PFOA, PFNA, and PFOSA) were detected in human milk samples. Two of them, PFOS and PFHxS, were found in all samples at mean concentrations of 201 and 85 ng/L, respectively. At the time in which that study was conducted, only the results of a previous survey on the concentrations of various PFC in human milk of China had been reported (So et al. 2006). The results obtained by Kärrman et al. (2007) were comparable to those of So et al. (2006), excepting those for PFOSA, a compound not included in the Chinese study, in which other PFC (PFHpA, PFDA, and PFUnDA) was also detected. The estimated total PFC transferred by lactation to a breastfed infant was found to be 200 ng/day (Kärrman et al. 2007). In another Swedish study, samples of human milk were collected in Stockholm and Gothenburg during the periods 1972–2016 and 2007–2015 (Nyberg et al. 2018). The inter-individual, inter-city, and temporal trends of several PFAS (PFOS, PFOA, FOSA, branched perfluorooctanesulfonamide (Br-FOSA), linear perfluorooctanesulfonamide (L-FOSA), PFBS, PFDA, PFDoDA, PFHpA, PFHxA, PFHxS, PFNA, branched perfluorooctane sulfonate (Br-PFOS), linear perfluorooctane sulfonate (L-PFOS), PFTeDA, perfluorotridecanoic acid (PFTrDA), PFUnDA, methylperfluorooctanesulfonamidoacetic acid (MeFOSAA), and ethylperfluorooctanesulfonamidoacetic acid (EtFOSAA)) were investigated. In general, the levels, profiles, and inter-individual variability were similar in the samples of both cities. The profiles were dominated by PFOS and PFOA, whose mean levels were 53 ± 27 and 53 ± 24 ng/L, respectively. The ∑PFAS concentrations (lower and upper bound estimates: LB, UB) ranged from 83 to 290 ng/L (LB) and 110–310 ng/L (UB) for the samples of Stockholm, and 61–290 ng/L (LB) and 90–320 ng/L (UB) for those of Gothenburg. The EDIs (LB) for ∑PFAS levels in infants ranged between 7.1 and 40 ng/kg bw/day, and between 5.2 and 25 ng/kg bw/day, in Stockholm and Gothenburg, respectively. It was concluded that while exposure to some legacy PFAS via breast milk was declining, concentrations of other PFAS were increasing in recent years, which might mean potential health risks to infants. In a subsequent study, the same research group (Awad et al. 2020), which was joined by Chinese researchers, measured the concentrations of 20 PFAS in samples of human milk from China (Shanghai, Jiaxing and Shaoxing), being the results compared with those previously obtained by the Swedish group (Nyberg et al. 2018). While in the Swedish survey, PFOS and PFOA were the dominant compounds, the highest levels of PFAS in China corresponded to PFOA, 9-chlorohexadecafluoro-3-oxanonane-1-sulfonic acid (9Cl-PF3ONS) (trade name “F53-B”), and PFOS (up to 411, 976 and 321 ng/L). 9Cl-PF3ONS (also 11Cl-PF3OUdS) was not detected in the samples of the Swedish survey, which was not surprising considering that this product was produced/used exclusively in China. The mean Σ_20_PFAS EDIs in China were 66, 40, and 37 ng/kg bw/day in the cities of Jiaxing, Shanghai, and Shaoxing, respectively, being notably lower, 11 ng/kg bw/day, in Stockholm. The high levels of 9-Cl-PF3ONS found in Chinese samples suggested that if the assessment of human exposure to PFAS in breastfeeding infants is focused only on legacy substances, it could underestimate overall PFAS exposure. On the other hand, Sundström et al. (2011) examined the temporal trend (1972–2008) of the levels of PFOS, PFHxS, and PFOA in pooled human milk samples from mothers from Stockholm. In general, for most of the analyzed years, the highest levels corresponded to PFOS, whose average concentrations were about twice that of PFOA, and 11 times that of PFHxS. The highest concentrations of these compounds corresponded to the years 1997 for PFOS (237 ng/L), 1998 for PFHxS (28 ng/L), and 1995 for PFOA (139 ng/L). For these three compounds, increasing trends from 1972 through the late 1990s were observed, with a decline already noted in 2001, which continued until 2008. Specifically, the levels of PFOS, PFHxS, and PFOA detected in 1972 were 23, < 5, and 19 ng/L, while in 2008, the concentrations were 75, 14, and 74 ng/L, for PFOS, PFHxS, and PFOA, respectively. That was the first study focused on determining the levels of PFOS, PFHxS, and PFOA in human milk continuously, starting in 1972, the beginning of large-scale fluorochemical production, going through 2001, years of the major manufacture, and ending in 2008. The authors noted that that the temporal trend in the levels of PFOS, PFHxS, and PFOA in human milk samples was like that reported by other authors regarding serum concentrations of these same compounds.

In Finland, Lamichhane et al. (2021) conducted a study aimed at investigating the possible association between the concentrations of various PFAS (PFDA, PFHxS, PFNA, PFOA, Br-PFOS, and L-PFOS) in maternal blood and the lipidomic profile of breast milk. The influence on the intestinal immunomodulatory functions in the infant gut was also assessed. A lipidomic analysis of breast milk was carried out in samples collected 2–4 days after delivery, as well as at 3 months of infant age. The results suggested that exposure to PFAS decreased the nutritional quality markers of human milk, with reduced total lipid content. These changes were linked to altered infant growth and intestinal inflammatory markers. In summary, exposure to PFAS would affect the lipid composition of breast milk lipid, especially in mothers with infants prone to autoimmune diseases. This finding was corroborated in a recent study on the impact of environmental factors on human breast milk lipidome in future immune-mediated diseases, in which the impact of PFAS was examined (Hyötyläinen et al. 2024).

In the Faroe Islands, Mogensen et al. (2015) conducted a study to assess the role of breastfeeding as an exposure pathway for five PFAS (PFOS, PFOA, PFHxS, PFNA, and PFDA). Serum levels of these compounds were measured in a Faroese birth cohort at birth, and at 11, 18, and 60 months. It was observed that after cessation of breastfeeding, the serum concentrations in infants decreased, which would be an indirect indicator that breastfeeding is an important pathway of exposure to PFAS in infants. However, breast milk concentrations were not reported. The results of another study on the effects of exposure to PFAS during infancy were recently published by Grandjean et al (2023), in which serum adipokine concentrations at age 9 years were used as biomarker instead of serum-PFAS levels. The results suggested that early postnatal PFAS exposure could affect subsequent metabolic health. In that study, concentrations of PFAS in milk samples were not measured/reported.

#### Mediterranean European countries

Kadar et al. (2011) developed a quantitative method to measure PFC in human breast milk, which was used to analyze the concentrations of 14 PFC in 30 breast milk samples, collected at a regional scale, providing the first set of preliminary data regarding perinatal exposure in France. PFOS and PFOA were detected in all the analyzed samples, being the means 78 and 59 ng/L (ranges 24–171 ng/L and 18–102 ng/L), respectively. In contrast, PFBA could be quantified only in one sample, while the concentrations of the remaining PFC were below the respective limits of detection (LODs). In general, the data obtained for French population were in the same range than previously reported results for other countries. The same research group conducted another survey aimed at determining the concentrations of 14 PFAS (PFBA, PFPeA, PFHxA, PFHpA, PFOA, PFNA, PFDA, PFUnDA, PFDoDA, PFBS, PFHxS, PFHpS, PFOS, and PFDS) in 48 breast milk samples of French women (Antignac et al. 2013). Three PFAS, PFOS, PFOA, and PFHxS, were detected at high percentages: 90%, 98%, and 100%, respectively, PFBA was found in 17% of the samples, while PFNA, PFHxA, and PFHpA were detected only in one of the 48 analyzed samples. The rest of PFAS were not detected in any breast milk sample. The median (range) concentrations of PFOS, PFOA, and PFHxS were 79 (5–330) ng/L, 75 (< 50–220) ng/L, and 50 (40–79) ng/L, respectively. No significant relationships were noted between the exposure levels of PFAS and developmental outcomes.

In Italy, Guerranti et al. (2013) carried out a pilot study in which the distribution and concentrations of PFOS and PFOA in 49 milk samples (24 primiparae and 25 multiparae women) from the Sienese area (Tuscany, central Italy) were determined. PFOS was detected in 20 samples, with an average value of 0.88 ng/g wet weight (equivalent to 0.85 ng/mL; range 1.02–4.28 ng/mL). PFOA could be detected only in one sample from a primipara at a concentration of 7.78 ng/mL, while the estimated average—calculated across the total sample set—was 0.16 ng/mL. Based on these results, it was concluded that intake of breast milk would not result in health risks for infants, considering that the TDIs of PFOS and PFOA were not exceeded. In the databases used to prepare the current review article, two studies conducted in the University of Bologna are cited. Barbarossa et al. (2013) measured the concentrations of PFOS and PFOA in human milk from Italy, and assessed the potential correlations between the primipara/multipara status of the pregnant women and the concentrations of both PFC. Thirty-seven milk samples were collected. From these, 21 belonged to primipara women, being the rest of women nursing for, at least, a second time. PFOS was quantified in 90% of the samples of primiparous mothers (mean: 57 ng/L, range 15–288 ng/L), and in and 62% of women who had already breastfed previously (mean: 36 ng/L, range 15–116 ng/L). Regarding PFOA, it was quantified in 81% of cases in primiparas (mean: 76 ng/L, range 24–241 ng/L), and 46% of cases in multiparas (mean 43 ng/L, range 24–241 ng/L). It was concluded that the toxicological risks due to the intake of these pollutants through breastfeeding would be rather moderate. In a more recent study, Aceti et al. (2021) assessed the potential exposure of preterm infants to PFAS through human milk. The levels of four PFAS (PFOS, PFOA, PFHxS, and PFNA) in milk samples of 35 women (15 term, 10 preterm, and 10 donor) were measured, being the daily intake at full enteral feeding subsequently estimated. PFOA (58.7%) was the main contributor to total PFAS, followed by PFOS and PFNA (in a much lower percentage), being PFHxS practically absent. The sum concentrations of the analyzed PFAS were 135, 130, and 112 ng/L for the term, preterm, and donor groups, respectively. The median EDIs for total PFAS were 20.72 ng/kg bw/day (range 10.72–107.84) and 17.92 ng/kg bw/day (range 6.4–28.96) for the preterm and donor groups, respectively. It was concluded that exposure of preterm infants to PFAS through human milk belonging to the mothers (or to donors) could exceed reference values for older infants.

In Spain, the first data regarding the occurrence of PFC in human milk were published at the beginning of the past decade (Kärrman et al. 2010; LLorca et al. 2010). Kärrman et al. (2010) determined the concentrations of 12 PFC (PFHxS, PFOS, PFOA, PFNA, PFDA, PFBS, PFHxA, PFHpA, PFDoDA, PFTDA, PFUnDA, and 2-(perfluorohexyl)ethane-1-sulfonic acid (THPFOS)) in ten samples of milk of healthy primipara mothers, living in Tarragona County, Catalonia. Only PFHxS and PFOS were detected, with mean concentrations of 40 and 120 ng/L, respectively. The remaining ten PFC could not be detected in any sample. The results of that survey were, in general, like those reported for other countries. In turn, Llorca et al. (2010) measured the levels of six PFC (PFOA, PFOS, i,p-PFNA, PFNA, PFDA, and PFDS) in different types of childbirth food, including 20 human breast milk, and evaluated the Risk Index (RI) for daily childhood intake of these compounds. PFOS, i,p-PFNA, and PFOA were the most frequently detected PFC, with PFOS and i,p-PFNA being quantified in 95% of the samples and PFOA in 45% of the analyzed samples. The range of concentrations for these compounds were as follows: 28–865 ng/L, 21–260 ng/L, and < LOQ–907, for PFOS, i,p-PFNA, and PFOA, respectively. With respect to the RIs calculated for the first 6 months of life, the ingestion rates of PFOS and PFOA (excepting one sample) did not exceed the TDI recommended by the European Food Safety Authority (EFSA). On the other hand, Motas Guzmán et al. (2016) measured the levels of five PFCAs in 67 Spanish breast milk samples collected in the region of Murcia. The frequencies of detection ranged between 3% for PFDoDA and 60% for PFOA. The median (range) levels (ng/L) of the 5 analyzed PFCAs were the following: 26 (< LOQ–211), 40 (15–70), 20 (< LOQ–34), 20 (16–57), and 21 (16–26), for PFOA, PFNA, PFDA, PFUnDA, and PFDoDA, respectively, being 29 (< LOQ–397) ng/L the median (range) of the ΣPFCAs. When a comparison of the PFCAs concentrations was carried out between women nursing for the first time, with those who had nursed previously, the mean concentrations were 96 (range 13–397) ng/L, in the case of mothers lactating for the first time, and 40 (range 13–167) ng/L) in milk samples from multiparous. As in the previous study by Llorca et al. (2010), the EDI of PFOA, calculated for the first 6 months of life, did not exceed the TDI. In turn, Beser et al. (2019) developed an analytical strategy to determine in breast milk the occurrence and concentrations of PFAS and organophosphorus compounds (OPs). Analyses were conducted in 20 milk samples obtained from 14 women living in the region of Valencia. Among the 12 PFAS analyzed, only four could be quantified: PFPeA (detected in 19 samples; mean: 158 ng/L, range ND–176 ng/L), PFOA (detected in 17 samples; mean: 152 ng/L, range ND–180 ng/L), PFOS (detected in 11 samples; mean: 66 ng/L, range ND–78 ng/L), and PFNA (detected only in one sample, at 70 ng/L). PFHxS was found in six samples, but it could not be quantified. The concentration of total PFAS ranged between 66 and 356 ng/L. The daily intakes of the most known and investigated PFAS, PFOA and PFOS, were also estimated for two scenarios: medium and higher intake. For PFOA, these intakes (μg/kg bw/day) were 0.0021 and 0.0029, respectively, while for PFOS the EDIs were 0.0092 and 0.01254, respectively. All the EDIs were notably lower than the TDI for PFOA and PFOS. Recently, Serrano et al. (2021) evaluated the levels and profiles of 11 PFAS in milk samples obtained from donors in a human milk bank in Granada (SE Spain). The highest frequencies of detection corresponded to PFHpA (100%) with a median concentration of 19.39 ng/L, PFOA (84.1%, and median level of 7.17 ng/L), PFNA (70.7%, and median concentration of 2.59 ng/L), PFHxA (65.9%, and median level of 1.58 ng/L), and PFTrDA (62.2%, and median level of 1.69 ng/L). PFDA, PFUnDA, PFDoDA, PFBS, PFHxS, and PFOS were detected only in less than 40% of milk samples. The median concentration of the sum of all 11 analyzed PFAS was 87.67 ng/L. In 2020, the EFSA estimated that critical levels in breast milk would be 60 ng/L for PFOA and PFNA, and 73 ng/L for PFHxS and PFOS, being 133 ng/L the critical concentration for the sum of these four PFAS (EFSA 2020). The authors noted that the upper concentrations of their survey were comparable to the critical levels set by the EFSA. In a recent study, Rovira et al. (2022) determined the concentrations of various environmental pollutants in breast milk samples of a Spanish cohort, being the exposure of breastfed newborns to the analyzed contaminants also evaluated. Seven PFAS (PFOA, PFNA, PFDA, PFUnDA, PFHxS, PFHpS, and PFOS) were included in that survey. PFDA, PFHxS, and PFHpS could not be detected in any of the 60 breast milk samples, while the remaining compounds were found at the following percentages: PFOS (87%), PFNA (25%), PFOA (12%), and PFUnDA (5%), being their mean values 31, 6.8, 8.2, and 5.3 ng/L, respectively. These results were similar to those previously found in other Spanish studies (Kärrman et al. 2010, Llorca et al. 2010; Motas Guzmán et al. 2016; Serrano et al. 2021).

#### Other European countries

In Germany, Völkel et al. (2008) conducted the first study in that country specifically focused on evaluating the exposure of breastfed infants to PFOS and PFOA through human breast milk samples, in which the concentrations of both PFC had been previously determined. Milk samples were obtained as follows: 38 of them were archived samples from the breast milk bank of the University of Leipzig, while 19 were fresh milk samples from the neonatology ward of the University of Munich. In addition, 13 samples were derived from a study carried out in the previous decade at the children's hospital Gyor, Hungary. Thus, the concentrations of PFOS and PFOA were determined in 70 milk samples. PFOS was found in the 70 samples, being the median concentration 128 ng/L (range 28–639 ng/L). In contrast, PFOA could be quantified (LOQ: 200 ng/L) in only 16% of the samples, in a range between 201 and 460 ng/L. In general, the concentrations in the Hungarian samples were higher than those from Germany. For the German samples, the EDIs of PFOS via breast milk were 0.095 μg/day (using the median value) and 0.246 μg/day (using the maximum value), which indicated a low probability of exceeded the recommended TDI. As result of the first serious contamination by PFC in Germany, which occurred in 2006 in Sauerland (North Rhine-Westphalia), various studies aimed at assessing and managing that problem were carried out. Wilhelm et al. (2008) conducted a biomonitoring study in which the levels of PFOS and PFOA in 183 breast milk samples of women from that German region were measured. PFOS and PFOA were detected in 99 and 120 samples, with mean concentrations of 90 and 160 ng/L, respectively. The results suggested that fully breastfed infants would not exceed the allowable TDI (0.1 μg/kg bw/day) of PFC. In addition, the preliminary value of 540 ng/L (∑PFOS + PFOA in breast milk) was not exceeded. On the other hand, also in Germany, Fromme et al. (2010) performed an investigation aimed at improving the knowledge of exposure to PFC in utero and during critical periods of infant development. Samples of maternal blood, cord blood, blood from infants, and breast milk were collected from participants in Munich. Regarding specifically to breast milk samples, PFOS, PFOA, and PFHxS could be quantified at percentages of 72, 2, and 3%, respectively (on a total of 201 analyzed samples). The concentration ranges were < 30–110, < 150–250, and < 20–30 ng/L, for PFOS, PFOA, and PFHxS, respectively. The authors concluded that although the concentrations of PFC in breast milk were rather low, exposure to them through breastfeeding would explain the levels found in infants in the first 6 months, when almost all of them are exclusively breastfed. In another study of the same research group (Raab et al. 2013), the concentrations of PFOA, PFOS, and PFHxS were measured in 516 breast milk samples of Bavarian women, collected for conducting a wide study, in which various organochlorine compounds and nitro musks were also included. With respect to the PFAS, while PFOS could be quantified in 302 samples, PFOA and PFHxS were only quantified in seven and five samples, respectively. The mean (range) concentrations of PFOS, PFOA, and PFHxS were 60 ng/L (range < 20–260 ng/L), 80 ng/L (range < 80–290 ng/L), and 10 ng/L (range < 10–30 ng/L), respectively. The concentrations of PFOS were significantly higher in milk samples from women breastfeeding for the first time, than in those who had previously breastfed. The daily intakes for PFOS (medium intake: 0.008 μg/kg bw, and high intake: 0.021 μg/kg bw) and PFOA (medium intake: 0.006 μg/kg bw, and high intake: 0.025 μg/kg bw) were below the TDIs of 0.15 and 1.5 μg/kg bw estimated by the EFSA (2008). Even in the high-intake scenario, those intakes were only approximately 14% and 2% (PFOA) of the TDI values, for PFOS and PFOA, respectively.

In Belgium, Roosens et al. (2010) measured the concentrations of PFC, at different life stages, using pooled samples of human milk from various cities/regions of the country. For the analyzed PFC, the frequencies of detection followed this order: PFHxA > PFNA > PFOS ~ PFOA > PFDA > PFBS > PFHxS, while PFBA was not detected. For PFOS and PFOA, the median (range) concentrations were 2.9 (< 0.4–28.2) ng/mL and 0.3 (< 0.3–3.5) ng/mL, respectively, being the median ΣPFC, 7.7 (< 0.5–29) ng/mL. Interestingly, in that study the maximum levels of PFOS (28 ng/mL) and PFHxS (5.3 ng/mL) were among the highest values reported at that time in the scientific literature. In the Czech Republic, Cerna et al. (2020) measured the concentrations of 23 PFAS in samples of milk belonging to Czech breastfeeding primiparas, living in large agglomerations, industrial cities, smaller towns, and rural municipalities. To assess the temporal trends, samples were collected in four time periods (2006, 2010/2011, 2014, and 2017), being the number of milk samples 46, 183, 164, and 232 for each of these four sampling periods. The potential health risks for breastfed infants were also evaluated. Only PFOS and PFOA could be quantified in more than 90% of samples, being their median concentrations 75, 59, 35, and 23 ng/L for PFOA, and 45, 31, 29, and 20 ng/L for PFOS, respectively, in the sampling years 2006, 2010/11, 2014, and 2017. In turn, in the 2017 sampling, PFNA could be quantified in 99% of milk samples with a median concentration of 7 ng/L. For both, PFOA and PFOS, a significant downward trend with time was noted. The EDIs of PFOS and PFOA from breastfeeding were clearly lower than the TDI for both compounds set by the EFSA (2008). However, the comparison of PFAS intake with the provisional tolerable weekly intake (PTWI) would mean a potential increased risk for infants. In Ireland, Abdallah et al. (2020) determined the levels of ten PFAS in 16 pooled samples of milk from primiparas obtained at two maternity hospitals of Dublin. Only PFOA (100% of samples), PFNA (69%), PFHxS (31%), and PFOS (62%) were detected, being their median concentrations 100, 14, < 40, and 20 ng/L, respectively. In contrast, the concentrations of MeFOSAA, ethylperfluorooctanesulfonamidoethanol (EtFOSE), methylperfluorooctanesulfonamidoethanol (MeFOSE), FOSA, EtFOSAA, and PFBS, were all under the LODs (< 50–100 ng/L). In that study, the exposures of a one-month nursing infant to the detected PFAS through the human milk were estimated to be 18, 2.1, 3.5, and 2.4 ng/kg bw/day, for PFOA, PFHxS, PFOS, and PFNA, respectively, being below than the tolerable weekly intakes (TWIs) set by the EFSA (2008) for PFOA and PFOS.

The results of recent (2018–2024) European studies in which the concentrations of PFAS were determined in human milk samples are summarized in Table 2.

**Table 2:** A summary of recent (2018–2024) European studies in which the concentrations of PFAS in human milk samples were determined

Country/region	PFC/PFAS	Occurrence/ concentrations of PFOS and PFOA	Occurrence (detection frequency, DF)/ concentrations of other PFC/PFAS	Daily intakes of PFC/PFAS through milk by breastfeeding infants	Reference
Norway	PFOS, PFOA	PFOS: mean 126.70 (range 22.99–370.63) ng/L and PFOA, mean: 57.60 (range 2.19–182.58) ng/L	Other PFAS were not included in the study	Not reported	Iszatt et al. (2019)
Sweden (Stockholm and Gothenburg)	19 PFAS	Means ± SD: PFOS, 53 ± 24 ng/L and PFOA, 53 ± 27 ng/L	FHpPA was found in 8 samples from Stockholm at concentrations up to 42 ng/L, but only in one sample from Gothenburg (15 ng/L)	The EDIs (LB) for ∑PFAS levels in infants ranged between 7.1 and 40 ng/kg bw/day, and between 5.2 and 25 ng/kg bw/day, in Stockholm and Gothenburg, respectively	Nyberg et al. (2018)
Sweden (Stockholm and Gothenburg) and China (Shanghai, Jiaxing and Shaoxing)	20 PFAS	PFOS: 65 ± 22 ng/L (range 16−177 ng/L) PFOA: 139 ± 36 ng/L (range 64–308 ng/L)	In addition to PFOA and (PFOS), among all PFAS 9-chlorohexadecafluoro-3-oxanone-1-sulfonic acid (‘F53-B’) occurred at the highest concentrations. F53-B was detected only in Chinese cities	The mean Σ_20_PFAS EDIs were 66, 40, and 37 ng/kg bw/day for samples of Jiaxing, Shanghai and Shaoxing, respectively, being notably lower in Stockholm, 11 ng/kg bw/day	Awad et al. (2020)
Finland	PFDA, PFHxS, PFNA, PFOA, Br-PFOS and L-PFOS	Br-PFOS: 5.93 ng/mL (2.04–15.12 ng/mL; Detection Frequency (DF): 100%) L-PFOS: 7.24 ng/mL (range 3.02–19.48 ng/mL; DF: 100%) PFOA: 4.74 ng/mL (range 1.22–11.33 ng/mL; DF: 97%)	PFHxS: 0.23 ng/mL (range 0.15–0.38 ng/mL; DF: 100%) PFNA: 1.58 ng/mL (range 0.24–5.31 ng/mL; DF: 70%)	Not reported	Lamichhane et al. (2021)
Italy	PFOS, PFOA, PFHxS, and PFNA	PFOS: Term: 47 ng/L (IQR: 41–85 ng/L); Preterm: 34 ng/L (IQR: 22–178 ng/L); Donor: 26 ng/L (18–33 ng/L) PFOA: Term: 87 ng/L (77–115 ng/L); Preterm: 90 ng/L (68–108 ng/L); Donor: 72 ng/L (62–92 ng/L)	PFNA: Preterm: 6 ng/L (0–12 ng/L); Donor: 20 ng/L (0–24 ng/L)	The calculated median EDI for total PFASs was 20.7 ng/kg bw/day (range 10.7–107.8) for preterm human milk and 17.9 ng/kg bw/day (range 6.4–29.0) for donor human milk. In turn, median term EDI was 21.6 ng/kg bw/day, with a range of 9.3–97.2 ng/kg bw/day	Aceti et al. (2021)
Spain	PFHxA, PFHpA, PFOA, PFNA, PFDA, PFUnDA, PFDoDA, PFTrDA, PFBS, PFHxS, PFOS	PFOS: < 0.86 ng/L; DF: 34.1% PFOA: 7.17 ng/L; DF: 84.1%	PFHxA: 1.58 ng/L; DF: 65.9% PFHpA: 19.39 ng/L; DF: 100% PFNA: 2.59 ng/L; DF: 70.7% PFDA: < 0.72 ng/L; DF: 24.4% PFUnDA: < 0.74 ng/L; DF: 39% PFDoDA: < 0.77 ng/L; DF: 35.4% PFTrDA: 1.69 ng/L; DF: 62.2% PFBS: < 0.80 ng/L; DF: 35.4% PFHxS: < 0.66 ng/L; DF: 24.4%	Not reported	Serrano et al. (2021)
Spain	PFOA, PFNA, PFDA, PFUnDA, PFHxS, PFHpS and PFOS	PFOA: 8.2 ± 12 ng/L (range < 10–86 ng/L; DF: 12%) PFOS: 31 ± 18 ng/L (range < 10–76 ng/L; DF: 87%)	PFNA: 6.8 ± 3.6 pg/mL (range < 10–25 pg/mL; DF: 25%) PFUnDA: 5.3 ± 1.2 pg/mL (range < 10–11 pg/mL; DF: 5%)	PFOA and PFNA: 0.8 ng/kg bw/day (P50) PFUnDA: 0.6 ng/kg bw/day (P50) PFOS: 3.3 ng/kg bw/day (P50)	Rovira et al. (2022)
Czech Republic	23 PFAS	PFOA: 75 ng/L (2006); 59 ng/L (2010/11), 35 ng/L (2014); 23 ng/L (2017) PFOS: 45 ng/L (2006); 31 ng/L (2010/11); 29 ng/L (2014); 20 ng/L (2017)	PFNA: 7 ng/L (2017)	PFOA: 10 ng/kg bw/day (2006); 7.73 ng/kg bw/day (2010/11); 4.69 ng/kg bw/day (2014); 3.09 ng/kg bw/day (2017) PFOS: 6.00 ng/kg bw/day (2006); 4.00 ng/kg bw/day (2010/11); 4.08 ng/kg bw/day (2014); 2.65 ng/kg bw/day (2017) PFNA: 0.89 ng/kg bw/day (2017)	Černá et al. (2020)
Ireland	10 PFAS	PFOA: 130 ng/L (range 16–350 ng/L; DF: 100%) PFOS: 38 ng/L (range < 20–120 ng/L; DF: 62%)	PFHxS: < 40 ng/L (range < 40–87 ng/L; DF: 31%) PFNA: 26 ng/L (range < 10–100 ng/L; DF: 69%)	PFOA: 18 ng/kg bw/day PFHxS: 2.1 ng/kg bw/day PFOS: 3.5 ng/kg bw/day PFNA: 2.4 ng/kg bw/day	Abdallah et al. (2020)

### America

#### United States

Using in PubMed “perfluorinated compounds (PFC) in human milk” as search term, the first paper cited corresponds to Kuklenyik et al. (2004). These authors developed a method for measuring trace levels of 13 PFC in samples of milk and serum. While the concentrations of PFC in the analyzed blood samples were given, the results regarding human milk were not reported. Anyhow, it was suggested that PFC might not be as prevalent in milk as they were in serum. The first study reporting the occurrence and levels of PFC in breast milk from USA was carried out by Tao et al. (2008b). In the State of Massachusetts, these researchers collected 45 human milk samples, in which the concentrations of nine PFC (PFOS, PFOA, PFHxS, PFNA, PFHpA, PFDA, PFUnDA, PFDoDA, and PFBS) were measured. The daily intake of PFC in breastfed infants was subsequently estimated. PFOS and PFOA were the most detected, with percentages of 100% and 98%, respectively, while PFHxS and PFNA were found in 93% of the samples. The mean values of these 4 PFC were 131, 43.8, 14.5, and 7.26 ng/L, respectively. The rest of the analyzed compounds were detected only in a few samples. The mean EDI of total PFC by infants was estimated to be 23.5 ng/kg bw/day, being 87.1 ng/kg bw/day the highest intake. In a subsequent study, von Ehrenstein et al. (2009) measured the concentrations of nine PFC (PFOS, PFOA, PFNA, PFHxS, PFOSA, 2-(*N*-Methyl-perfluorooctane sulfonamido) acetic acid (Me-PFOSA-AcOH), 2-(*N*-Ethyl-perfluorooctane sulfonamido) acetic acid (Et-PFOSA-AcOH), PFBS, and perfluorodecanoate) in serum and milk samples of 34 breastfeeding women in North Carolina. PFC concentrations were lower than the respective limits of quantification (LOQs) set between 150 and 600 ng/L. Consequently, the partition coefficient from serum to milk could not be calculated. However, it was stated that milk concentrations were notably lower than serum concentrations. On the other hand, Zheng et al. (2021) analyzed 39 PFAS (nine short-chain PFAS and 30 long-chain PFAS) in 50 samples of breast milk collected from mothers. Sixteen of these PFAS were detected in 4–100% of the samples, with a ∑PFAS concentration ranging from 52 to 1850 ng/L (median concentration of 121 ng/L). PFOS and PFOA were the predominant compounds (medians of 30 and 14 ng/L, respectively). Nevertheless, they were generally lower than those found in breast milk samples from United States. The authors highlighted the fact that although PFOS and PFOA had been declining, occurrence of emerging short-chain PFAS had been increasing in the last years.

#### Canada

Kubwabo et al. (2013) developed methods for the extraction and determination in human milk of these compounds: five perfluorinated carboxylic acids (PFHxA, PFHpA, PFOA, PFNA, and PFDA), two perfluorinated sulfonates (PFHxS and PFOS), and eight polyfluorinated disubstituted phosphate surfactant congeners (4:2, 4:2/6:2, 6:2, 6:2/8:2, 8:2, 8:2/10:2, 10:2, and 10:2/12:2 diPAPS). Only PFOA was detected at a high percentage (85%) in the human milk samples analyzed, while most compounds could not be detected at concentrations above the method detection limits. The mean concentration of PFOA was 250 ng/L (range ND–520 ng/L). In turn, four diPAPS were detected and quantified in the milk samples at different concentrations, which ranged between < 10 and 830 ng/L.

#### Africa

To the best of our knowledge, there are only a couple of studies conducted in Africa on the topic of this review, or at least cited in PubMed or Scopus. Müller et al. (2019) evaluated prenatal exposure to various POPs (including six PFAS) in Tanzanian infants. The distribution of the measured POPs between breast milk, maternal and cord blood, and placenta and cord blood was also assessed. Samples were collected from 150 healthy primiparous mothers at a hospital in Arusha. However, data on the levels of PFAS in breast milk were not specifically reported. In turn, Macheka et al. (2022) analyzed the concentration of 15 PFAS in breast milk of nursing mothers from South Africa. The median concentrations of individual PFAS ranged between < LOQ and 0.730 ng/L, while the median levels of Σ_15_ PFAS ranged from < LOQ to 420 ng/L. While short-chain PFAS contributed to 55% of the mean concentration, PFOA and PFUnDA were detected in 94% of the samples. The EDIs ranged from 0.11 to 81.27 ng/kg bw/day, and 0.21 to 151.38 ng/kg bw/day for average and high-volume consuming infants, respectively. The EDI for PFOS was lower than the TWI for average consuming infants. In turn, HQ was below the unity, indicating negligible risk, while that of PFOA and ∑_4_ PFAS (PFOS, PFOA, PFNA, and PFHxS) presented considerable risk for breastfeeding babies.

## Discussion and conclusions

As stated in the introduction, blood is the most widely accepted and accurate medium for biomonitoring most POPs in general, and for PFC/PFAS in particular. In turn, breast milk has been often used as in biomonitoring studies carried out to detect environmental contaminants, being a vehicle for the accumulation of lipophilic and persistent substances, such as PFC/PFAS (Arendt 2008; Iribarne- Durán et al. 2022). In recent years, it has been shown that there is close relationship between the levels of certain PFAS (mainly PFOS and PFOA), in human blood of non-occupational exposed individuals and the concentrations in the drinking water consumed by those individuals (Wilhelm et al. 2009; Domingo and Nadal 2019; Pitter et al. 2020; Lahne et al. 2024). Similarly, the highest levels of PFAS of breast milk found in some of the above revised studies could be attributed to maternal exposure through contaminated drinking water, considering that drinking water can be a major exposure pathway in highly contaminated areas (Gyllenhammar et al. 2021; Mogensen et al. 2015). In April 2024, the US EPA announced Maximum Contaminant Levels (MCLs) for six PFAS in drinking water. PFOA and PFOS are now limited to 4 ppt, whereas PFNA, PFHxS, and HFPO-DA are limited to 10 ppt (US EPA 2024). In turn, some European countries have also opted to adopt stricter limits on the total concentration of PFAS-4 (PFOA, PFOS, PFNA, and PFHxS), aligning more closely with USA standards. Examples include Denmark (2 ppt), Sweden (4 ppt), and Germany (20 ppt, effective by 2028) (Gage et al. 2024).

The importance of breast milk, along with blood, relies on the fact that it reflects the mother’s exposure to these compounds through the diet and the environment. Altogether, it makes breast milk an invaluable tool to assess exposure to vulnerable populations, like lactating babies. Breastfeeding occurs in a critical developmental stage when the exposure to potentially toxic compounds like PFAS could have long-term implication for the infants, since organ, hormonal system, or nervous system are maturing (Lehmann et al. 2014). Therefore, in this context, biomonitoring is essential to understand the extent of exposure in the first years of life. Thus, measuring the presence of PFAS in breast milk not only provides information about maternal exposure but also offers valuable data on the transfer of these compounds from mother to infant. At the same time, it should help authorities implement more effective public health policies and preventive measures to protect children's health.

PFC/PFAS, which have been widely used in industries such as non-stick cookware, water-repellent clothing, and firefighting foams, are highly persistent in the environment and in living organisms, making them a long-term public health concern (Glüge et al. 2020; Meegoda et al. 2020). With respect to human exposure to these environmental pollutants, studies conducted in various countries have shown that certain regions experience high levels of these compounds, particularly PFOA and PFOS. In fact, in some regions of China, the HI was greater than the unity (at least for some samples), while other studies found that women from those areas had higher burdens than women from other regions and countries (So et al. 2006; Xu et al. 2022). Although there are more than 4000 different PFAS, there is only regulation for maximum levels in certain foodstuffs (and drinking water), and only for some of them, PFOA, PFOS, PFHxS, and PFNA, as well as the sum of these four compounds (Commission Regulation (EU) 2023/915) (EC 2023, 2024). Recently, the EFSA established a TWI of 4.4 ng/kg bw for the sum of PFOA, PFNA, PFHxS, and PFOS (EFSA 2020) meaning that the previous individual TDI for PFOS (150 ng/kg bw) and for PFOA (1.5 µg/kg bw) (EFSA 2008) should not be longer used as a toxicological threshold for exposure estimation. Most of the studies here reviewed were conducted before the implementation of the new TWI, suggesting all of them a low health concern for breastfeeding infants, even in a high-intake scenario (Kang et al. 2016; Guerranti et al. 2013; Llorca et al. 2010; Motas-Guzmán et al. 2016; Beser et al. 2019; Völkel et al. 2008; Wilhelm et al. 2008; Raab et al. 2013; Cerna et al. 2020). However, recently Reinikainen et al. (2024) reported that EU’s regulatory PFAS thresholds were impractical and with inconsistencies in the current risk-based approaches to PFAS. Outlooks for a potential improvement were suggested. Certainly, new studies should be carried out to check whether the EDIs are exceeding the current TWI. In this sense, the European Commission recommended Member States to monitor the presence of PFAS in food from 2022 to 2025 (EC 2021, 2023).

On the other hand, long-chain perfluorinated carboxylic acids (C9–C21 PFCAs) are being considered for inclusion in the Stockholm Convention, which will conclude in a global elimination, while PFHxA will face restrictions starting April 2026 (EC 2024). In addition, C9–C14 PFCAs have been restricted since February 2023 (Commission Regulation (EU) 2021/1297) (EC 2021). Due to the stricter regulation, in some regions and countries, particularly in Europe and North America, there has been a gradual decrease in the levels of legacy PFAS (Sunderland et al. 2019; Nyberg et al. 2018; Sundstrom et al. 2011; Černá et al. 2020). However, emerging PFAS, which are used as substitutes of legacy PFAS and do not have regulation, are becoming dominant in the environment (Li et al. 2020). Therefore, focusing only on legacy PFAS to calculate the exposure to these compounds would mean an underestimation of their exposure (Awad et al. 2020).

Throughout the twenty-first century, various authors have reviewed the state of the art regarding the concentrations of PFC/PFAS in human milk. Fromme et al. (2009) provided an early review on biomonitoring human exposure to PFC, discussing data on their concentrations in breast milk, blood, and human tissues within the general population of Western countries. In turn, Macheka-Tendenguwo et al. (2018) summarized data on PFAS in human breast milk, highlighting analytical challenges and the potential transfer pathways from maternal blood to milk. Similarly, Jian et al. (2018) reviewed studies on PFAS concentrations in various human tissues, including milk, and examined their distribution patterns. Focusing specifically on Arctic populations, Abass et al. (2018) presented an overview of temporal trends in environmental pollutants, including PFAS, and their health effects, using data from the Arctic Monitoring and Assessment Programme (AMAP) and Russian scientific literature. Breast milk was among the biological matrices reviewed by Abass et al. (2018). VanNoy et al. (2018) examined the relationship between PFAS exposure and breastfeeding, concluding that lactation served as a significant excretion pathway for PFAS in women, being human milk a critical exposure route for infants. In another review, Liu et al. (2020) explored pre- and neonatal exposure to PFAS across various biological matrices, such as umbilical cord blood, placenta, and fetal organs, with breast milk being one of the key matrices examined. Focusing on China, Hu et al. (2021b) reviewed studies assessing the concentrations and profiles of persistent organic pollutants (POPs), including PFAS, in human breast milk. That review evaluated potential health risks for breastfed infants under six months of age. Under the global monitoring plan (GMP) established by the Stockholm Convention on POPs, ambient air, human milk, blood, and water are key matrices for assessing the temporal and spatial distribution of POPs. Fiedler and Sadia (2021), Fiedler et al. (2022), and van der Veen et al. (2023) conducted reviews on PFAS concentrations in human milk, consistently identifying PFOS as the predominant PFAS. In Brazil, Barbosa Machado Torres et al. (2022) reviewed the occurrence of PFAS (listed as POPs under the Stockholm Convention), finding PFOS to be the most prevalent in terms of both, concentration and frequency. Recently, LaKind et al. (2023) provided a global comparison of PFAS levels in breast milk and infant formula, juxtaposed against health-based drinking water screening values for infants. It was concluded that the levels of PFOA and PFOS in breast milk frequently exceeded the screening values, regardless of geographical location.

Anyhow, the above reviewed studies suggest a decline in the concentrations of legacy PFAS (e.g., PFOS, PFOA) in breast milk in Europe and North America, which would be attributed to stricter regulations and reduced use of these compounds​. However, in some regions, concentrations of PFAS like PFOA have remained stable, or have even shown an increase over time, despite regulatory efforts. On the other hand, in recent years there is growing detection of emerging PFAS, which indicates a shift in exposure patterns due to the replacement of legacy PFAS. As happens with the levels of PFAS in blood samples, temporal trends in the concentrations of PFAS in human milk differ notably across regions and countries, reflecting local industrial activities, regulatory measures, and environmental conditions. Overall, the findings of this review underscore breast milk’s dual role as a reflection of maternal exposure and a pathway for infant exposure, urging continued and expanded monitoring and regulation.

## Data Availability

Data are available from the authors on request.

## Abbreviations

6:2 Cl-PFESA: 6:2 Chlorinated polyfluorinated ether sulfonate
6:2 diPAP: 6:2 Fluorotelomer phosphate diester
8:2 FTOH: 1H,1H,2H,2H-Perfluoro-1-decanol
8:2 FTCA: 8:2 Fluorotelomer carboxylic acid
8:2 FTUCA: 8:2 Fluorotelomer unsaturated carboxylic acid
9Cl-PF3ONS: 9-Chlorohexadecafluoro-3-oxanonane-1-sulfonic acid
10:2 FTOH: 1H,1H,2H,2H-Perfluoro-1-Dodecanol
C9-C11 PFCAs: Long-chain perfluorocarboxylic acids
ADHD: Attention-deficit/hyperactivity disorder
Br-FOSA: Branched perfluorooctanesulfonamide
Br-PFOS: Branched perfluorooctane sulfonate
EDI: Estimated daily intake
EFSA: European Food Safety Authority
EtFOSAA: Ethylperfluorooctanesulfonamidoacetic acid
EtFOSE: Ethylperfluorooctanesulfonamidoethanol
Et-PFOSA-AcOH: 2-(*N*-Ethyl-perfluorooctane sulfonamido) acetic acid
GMP: Global monitoring plan
HI: Hazard index
LB: Lower bound
LC-HRMS: Liquid chromatography coupled to high resolution mass spectrometry
L-FOSA: Linear perfluorooctanesulfonamide
LOD: Limit of detection
LOQ: Limit of quantification
L-PFOS: Linear perfluorooctane sulfonate
MCL: Maximum Contaminant Levels
MeFOSAA: Methylperfluorooctanesulfonamidoacetic acid
MeFOSE: Methylperfluorooctanesulfonamidoethanol
Me-PFOSA-AcOH: 2-(*N*-Methyl-perfluorooctane sulfonamido) acetic acid
OCP: Organochlorine pesticides
PBDE: Polybrominated diphenyl ethers
PCB: Polychlorinated biphenyl
PCDD/Fs: Dioxins and furan
PFAS: Per- and polyfluoroalkyl substances
PFBA: Perfluorobutanoic acid
PFBS: Perfluorobutanesulfonic acid
PFC: Perfluorinated compounds
PFCA: Perfluoroalkyl carboxylic acids
PFDA: Perfluorodecanoic aci
PFDoDA: Perfluorododecanoic acid
PFDS: Perfluorododecane sulfonate
PFHpA: Perfluoroheptanoic acid
PFHpS: Perfluoroheptanesulfonic acid
PFHxA: Perfluorohexanoic acid
PFHxS: Perfluorohexanesulfonic acid
PFNA: Perfluorononanoic acid
PFOA: Perfluorooctanoic acid
PFOS: Perfluorooctane sulfonate
PFOSA: Perfluorooctanesulfonamide
PFPeA: Perfluoropentanoic acid
PFSA: Perfluorosulfonic acids
PFTeDA: Perfluorotetradecanoic acid
PFTrDA: Perfluorotridecanoic acid
PFUnDA: Perfluoroundecanoic acid
POP: Persistent Organic Pollutant
PPAR: Peroxisome proliferator-activated receptors
PTWI: Provisional tolerable weekly intake
ROS: Reactive oxygen species
SPE: Solid-phase extraction
TDI: Tolerable daily intake
THPFOS: 2-(Perfluorohexyl)ethane-1-sulfonic acid
TWI: Tolerable weekly intake
UB: Upper bound
